# Better Training for Matrons

**Published:** 1909-01-23

**Authors:** 


### Nursing Administration.

### BETTER TRAINING FOR MATRONS.

### III.—LEARNING AND TEACHING.

For the first two years of training no differentiation between probationers can well be attempted. Even although the lessons given in certain subjects may appear to the eager pupil below the level she might reasonably reach, it is desirable that she who will hereafter have to direct the studies of other nurses should learn the rudiments of her business in the average way among average companions. There is so much to which the pupil must accommodate her mind during the first experience of hospital life that this period ought not to be overweighted with book work, and the more simply the elements of nursing can be presented to the beginner, the better she will be adapted for putting others through the mill hereafter. At the end of two years the probationer, though still in many training schools retaining that name, is in truth something more. She is of great and increasing value to the hospital, and is qualified if her training has proceeded on good lines, not only to do most of the duties required of a nurse, but even to act on occasion as charge nurse and to superintend others. This is the point at which, if her character and talents mark her out as suitable, she might proceed to a further course of study, with the view to fitting herself for the higher administrative posts in the future. Study is easier at this stage than in the first crowded months of hospital experience. Assuming the entrance age to be 23, the probationer is now 25, and she ought to be able to continue her studies under direction without the necessity for detailed lectures on every point. The difficulty of arranging formal courses of lectures, which all the students must be set free to attend at a stated hour, would be considerably lessened by the plan of setting subjects for private study and testing by means of papers and questions the individual students in their progress. In this way preparation could be given for the examinations of the Science and Art Department in Physiology and such other subjects as should be decided on. To possess the requisite information is but one step in the knowledge of the subjects required by a woman who aspires to the post of matron. It is facility in imparting the knowledge which will prove most useful to her in the future. For knowledge which has grown rusty from disuse can always be rubbed up with the aid of good text-books, but the matron who has never had any practice in teaching will always be at a disadvantage. During the time, then, that the advanced curriculum is proceeding, one special feature should be the constant preparation of the students for giving systematic instruction by setting them to coach the junior probationers, and so by degrees to take classes and give lectures themselves. The lessons should from time to time be given in the presence of the matron or sister who is conducting this part of the course, and there should be free criticism afterwards. The student should, moreover, be exercised in giving practical demonstrations to the juniors, and her methods of giving directions for the right performance of such duties as bed-making, bandaging, washing a dummy patient, etc., should be freely criticised in the absence of the pupils. Since it is one of the principal duties of everyone in the training school to be instructing, such demonstrations would enter into the regular routine of the hospital, but instead of the instruction being done after the rule of thumb, with no attention to the method of teaching so long as the pupil is made to understand in the end, each nurse who desired to remain in institution work would be trained by constant practice and expert advice how to make her teaching tell, how to make her pupils efficient with the smallest amount of time and talk. It may be said that much of this goes on already in a well-ordered hospital. True this kind of help is readily accessible in the ordinary course of work, and for that reason it could easily be made accessible for all who want to learn, and drawn into line as part of the system. Many a woman is trained in a big hospital and becomes herself in time a matron without once having any insight given her into the business of coaching and the difficulties of dealing with slow or awkward probationers. She falls back on her own experiences when she began to learn, and it is this which keeps the general art of teaching at a low level. It may then be stated generally that whatever standard of knowledge the matron's curriculum should embrace, the time spent during these years of study should be pretty equally divided between learning and teaching. In this way alone can the student's knowledge be at her disposal whenever it is wanted. A profound insight into the mysteries of bacteriology cannot be expected from a woman who is engrossed for the greater part of the day in anxious and responsible duties. But what she knows she ought to know clearly and with due reference to the depths of the subject out of sight. She ought to be capable of putting the facts concisely at a moment's notice to pupils less clear-headed than herself and of showing their practical application to the duties in hand. But none of this comes by nature, and she ought to be taught how to marshal her facts and express them in clear forcible language, for it is this which will avail her as head of a training school. In examining students for a certificate of competence to perform the duties of a matron, this trained habit of instruction would naturally take an important place. She would be expected to show that she could give a lecture on elementary physiology or on some nursing subject, and also that she could give a practical demonstration, satisfying the examiners in method and mode of expression. Experience shows that when marks are assigned to any particular branch of a subject in the final examination, the general level of attainment in that branch is raised right through the establishment. It is only because they are not tested by the examiner that the methods adopted by sisters and charge nurses in instructing young probationers continue to leave so much to be desired.

